# Continuous-wave quantum dot photonic crystal lasers grown on on-axis Si (001)

**DOI:** 10.1038/s41467-020-14736-9

**Published:** 2020-02-20

**Authors:** Taojie Zhou, Mingchu Tang, Guohong Xiang, Boyuan Xiang, Suikong Hark, Mickael Martin, Thierry Baron, Shujie Pan, Jae-Seong Park, Zizhuo Liu, Siming Chen, Zhaoyu Zhang, Huiyun Liu

**Affiliations:** 1 School of Science and Engineering, The Chinese University of Hong Kong, 518172 Shenzhen, Guangdong P.R. China; 2 Department of Electronic and Electrical Engineering, University College London, Torrington Place, London, WC1E 7JE UK; 3 Univ. Grenoble Alpes, CNRS, CEA-LETI, MINATEC, LTM, F-38054 Grenoble, France

**Keywords:** Lasers, LEDs and light sources, Semiconductor lasers, Photonic crystals, Quantum dots, Semiconductor lasers

## Abstract

Semiconductor III–V photonic crystal (PC) laser is regarded as a promising ultra-compact light source with unique advantages of ultralow energy consumption and small footprint for the next generation of Si-based on-chip optical interconnects. However, the significant material dissimilarities between III-V materials and Si are the fundamental roadblock for conventional monolithic III-V-on-silicon integration technology. Here, we demonstrate ultrasmall III-V PC membrane lasers monolithically grown on CMOS-compatible on-axis Si (001) substrates by using III-V quantum dots. The optically pumped InAs/GaAs quantum-dot PC lasers exhibit single-mode operation with an ultra-low threshold of ~0.6 μW and a large spontaneous emission coupling efficiency up to 18% under continuous-wave condition at room temperature. This work establishes a new route to form the basis of future monolithic light sources for high-density optical interconnects in future large-scale silicon electronic and photonic integrated circuits.

## Abstract (web summary)

Fabricating semiconductor photonic lasers based on III-V materials are challenging because of the material mismatch with silicon. Here the authors monolithically grow quantum-dot-based photonic crystal membrane lasers directly on an on-axis silicon substrate.

## Introduction

The recent exponential growth in data traffic requires a more efficient on-chip optical interconnection method with lower energy consumption and higher density of processing unit^1,2^. The low-cost Si-based on-chip photonic networks integrated with nanoscale, high modulation speed and low-energy cost optical components have attracted much attention in past few decades, providing various potential applications in many areas especially short-distance optical communication within data centres^3–7^. Recently, integrating III–V lasers on CMOS-compatible Si platforms has been proved as the most efficient method to resolve the issue of Si’s indirect bandgap property, despite great efforts have been made on group-IV lasers^8^. Even though high-performance Fabry-Perot and distributed feedback lasers integrated on Si are extensively studied^9,10^, seeking a method to decrease the volume of the laser cavity and active region, and reduce the operating energy is another task to realise more energy-efficient Si-based photonic integrated circuits (PICs). In this regard, nanoscale PC cavity with high-quality-factor (*Q*-factor), ultrasmall mode volume (*V*_mode_) and large Purcell factor (proportional to *Q*/*V*_mode_) is one of the most promising architectures for integrated nanoscale devices, with the advantage of ultralow energy consumption as a result of enhanced light-matter interaction^11,12^. Extremely high light confinement near the ultimate volume limit *λ*/2*n* in all dimensions is achieved by both lateral confinements using distributed Bragg reflection and out-of-plane confinement based on total internal reflection. Currently, such high-*Q* nanocavities are the focus of much interest, and a number of leading-edge studies have been reported with various functional devices, including modulators^13–16^, memories^17,18^ and lasers^11,12,19^. Recently, high-speed and ultracompact PC laser sources with ultralow power consumption have been demonstrated^13,20–24^, which offers perspective light sources for the next-generation on-chip photonic integrated circuits.

Despite the great efforts that have been devoted on heterogeneous integration of PC lasers on Si^25–27^, the monolithic integration is the most promising approach for a higher yield, higher density and scalability for III–V PC lasers integrated on Si platforms, which will further increase density and yield of fabricated laser compared with III–V ridge-waveguide lasers and bonded III–V PC lasers on Si^28–30^. Moreover, the monolithic integration is an ideal solution to reduce the substrate cost by growing III–V materials on large-scale Si wafers, instead of using dedicated and expensive GaAs and InP wafers^31–34^. However, the major challenge of monolithic integration is the significant degradation of the crystal quality of metamorphic III–V layers on Si due to the large material mismatch in lattice constant, thermal expansion coefficients, as well as polarity^35–37^. Tremendous efforts have been made through the optimisation of III–V buffer layers and sophisticated epitaxial technologies to realise a low-defect-density and CMOS-compatible III–V/Si virtual substrate^37^. In addition, zero-dimensional materials—III–V quantum dots (QDs)—monolithically grown on Si platform as gain materials provide various advantages, including low lasing threshold, reduced temperature sensitivity^38^, and less sensitivity to defects, and hence have been widely investigated in past few years. Until now, high-performance III–V QD distributed feedback lasers, ridge-waveguide lasers, microring or microdisk lasers have been successfully demonstrated, all of which were epitaxially grown on Si, including off-cut (4°–6°) Si substrate^38,39^, patterned on-axis Si (001)^40–43^ and on-axis Si (001)^44–46^. However, monolithically integrated III–V PC lasers on the well-established Si CMOS fabrication technologies have not been demonstrated yet due to the high requirement of crystal quality. High optical loss and increased non-radiative recombination ratio make Si-based III–V PC laser difficult to be realised. Implementing defect-insensitive QD active region on the high crystal quality buffer layer is the key to achieve PC lasers monolithically grown on a Si substrate.

Here, we present InAs/GaAs QD PC membrane lasers monolithically grown on on-axis Si (001) substrates with optimised III–V buffer layers, for easily manufacturable on-chip Si light sources with dense integration and low power consumption. The ultrasmall PC laser with the theoretical mode volume *V*_mode_ (${\int} {\left( {\varepsilon E^2} \right)/\max \left( {\varepsilon E^2} \right)dr^3}$) of 0.88 (*λ*/*n*)^3^ is operated under optically pumped continuous-wave (CW) conditions at room temperature. Both an ultra-low lasing threshold of ∼0.6 µW and a large spontaneous emission coupling efficiency (*β*) 0.18 were obtained for a PC laser with lattice constant *a* = 310 nm and air-holes radius *r*/*a* = 0.27. The Si-based PC lasers presented in this paper, providing great advantages in terms of a small footprint as well as low power consumption, can be a promising light source in the next-generation nanoscale Si photonics.

## Results

### Epitaxial growth and optical characterisation of QD PC lasers

**Fig. 1:**
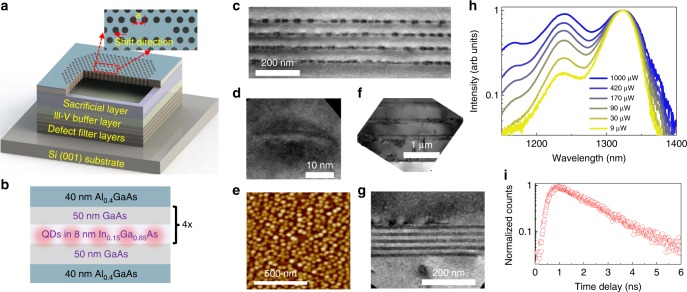
Epitaxial growth, structural and optical characterisation. **a** Schematic diagram of the fabricated InAs/GaAs QD PC (*L*3 cavity) laser epitaxially grown on on-axis Si (001) substrate. The lattice constant, radius and shift of *L*3 PC cavity are *a*, *r* and 0.15*a*, respectively. **b** Schematic epitaxial structure of active region for the PC laser. **c** and **d** show the high-resolution cross-sectional bright-field STEM images of the four-stack InAs QD layers and a single QD, respectively. **e** demonstrates an AFM image of uncapped InAs/GaAs QDs grown on Si (001) substrate. **f** presents a bright-field STEM image of the interface between defect filter layers and on-axis Si (001) substrate. **g** shows the high-resolution TEM image of a defect filter layers. **h** Logarithmic plot of the normalised collected PL spectra under various input power at room temperature. **i** Time resolved PL measurement of the as-grown structure at room temperature, indicating a radiative recombination lifetime around 1.58 ns.

3D finite-difference time-domain (FDTD) methods were used to optimise the structural parameters in order to obtain a high-*Q* resonance within the QD ground state gain spectrum (Supplementary Fig. 1). Figure 1a shows a schematic diagram of the fabricated InAs/GaAs QD *L3* defects PC lasers epitaxially grown on on-axis Si (001) substrates. The air slab (with a thickness of ∼1 µm) underneath the cavity enhances the light confinement in the vertical direction. The lattice constant, the radius of etched air-holes and a shift distance of the *L3* defects PC are denoted by *a*, *r* and 0.15*a*, respectively. Figure 1b illustrates the epitaxial structure of the active region, which consists of four-stack well-developed InAs/In_0.15_Ga_0.85_As dot-in-well (DWELL) layers separated by a 50-nm GaAs spacer layer and two symmetrical 40-nm-thick Al_0.4_Ga_0.6_As cladding layers. Figure 1c, d illustrate the high-resolution transmission electron microscope (TEM) images of the as-grown four-stack InAs/GaAs QD layers and a single QD, respectively. An atomic force microscope (AFM) image of uncapped InAs/GaAs QDs grown on Si (001) substrate is presented in Fig. 1e. Figure 1f shows a cross-sectional TEM image of the interface between GaAs buffers and Si (001) substrate, and the interface of InGaAs/GaAs strained-layer superlattice defect filter layers (DFL). A high-resolution cross-sectional TEM image of DFL presented in Fig. 1g indicates five layers of In_0.18_Ga_0.82_As/GaAs strained-layer superlattices. The InAs/GaAs QD density is estimated to be ∼4 × 10^10^ cm^−2^ with a typical size of 25 nm in diameter and 8 nm in height, determined by the AFM and TEM images shown in Fig. 1e and Fig. 1c, respectively. Room-temperature micro-photoluminescence (µ-PL) measurement of the as-grown structure was carried out to measure the emission spectra under various input power, as shown in Fig. 1h, which indicates that ground state emission was at ∼1.3 µm within O-band with a full width at half maximum of 28 meV. Band filling effect is evident with increasing the pumping power, which shows the first excited state centred at 1.24 µm and the second excited state as a shoulder at 1.16 µm. The well-resolved energy levels have an energy separation of 63 meV between the ground state and the first excited states, and 69 meV between the first excited and the second excited states. Time resolved PL measurement of the as-grown structure indicates a radiative recombination lifetime around 1.58 ns, as shown in Fig. 1i.

### 3D-FDTD simulation

**Fig. 2:**
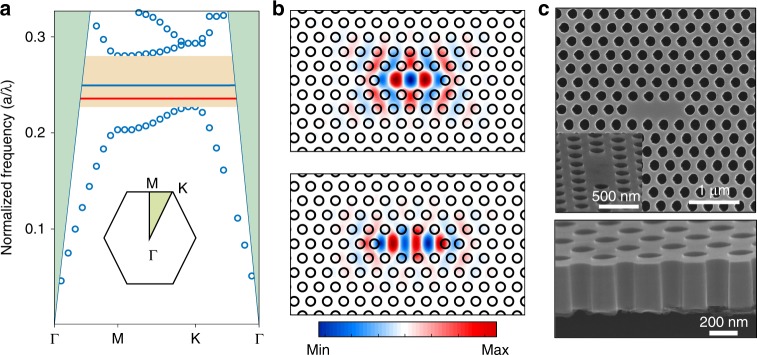
3D-FDTD simulation and PC cavity. **a** Fundamental TE mode band diagram of the triangular lattice PC cavity with the irreducible Brillouin zone, calculated by using 3D-FDTD method. The shaded green region is the light cone and the yellow region indicates the photonic band gap. **b** The calculated *E*_y_ field profiles of the fundamental mode and the higher order mode of the PC cavity, of which the normalised frequencies are presented as the red and blue line in **a**. **c** Top-view and tilted cross-section view SEM images of the fabricated PC cavity.

Figure 2a depicts the calculated fundamental transverse electric (TE) mode band diagram of the triangular lattice PC with *a* = 310 nm, *T* = 362 nm, *r* = 0.27*a* and refractive index of 3.4 by using the 3D-FDTD method. The shaded green region is the light cone. The yellow region in the band diagram represents the photonic band gap with normalised frequency (*a*/λ) from 0.225 to 0.275, and the blue and red lines in the band gap region indicate the normalised frequency positions of the fundamental mode and the first higher order mode under the same structural parameters, respectively, of which the electric field (*E*_y_) profiles are depicted in Fig. 2b. The fundamental mode within the *L3* defects PC cavity exhibits a much higher *Q*-factor than other higher order modes while keeping a small mode volume *V*_mode_ of 0.88 (*λ*/*n)*^3^ (Supplementary Fig. 1). During the experiments, the etching profiles of air-holes have a significant impact on the lasing wavelength and threshold. A smooth and vertical surface of air-holes is expected to reduce the lasing threshold. Figure 2c shows the top-view and tilted cross-section view SEM images of the fabricated PC cavity, and the inset illustrates a magnified view of the cavity region. Some residues still remain on the wet-etched undercut surface, as shown in the cross-section SEM image in Fig. 2c. These residues adhered to the undercut surface may reduce the *Q*-factor of the resonant modes due to increased optical scatter loss (Supplementary Fig. 2), and a clean undercut surface is expected by increasing the Al composition within the AlGaAs sacrificial layer^47^.

### PC laser performance characterisation

**Fig. 3:**
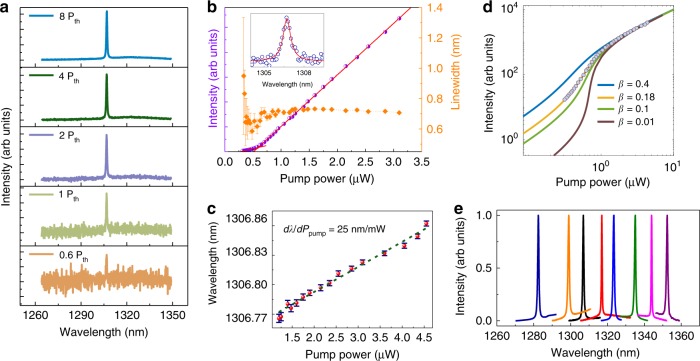
Laser performance characterisation. **a** Measured spectra under various input pumping powers of the PC laser with *a* *=* 310 nm and *r*/*a* *=* 0.27, the lasing peak locates within the ground state emission. **b** Collected *L*–*L* curve and linewidth of the lasing peak at 1306 nm, indicating a lasing threshold 0.6 μW. The inset shows Lorentzian curve fitting of measured data just below the threshold, which indicates a linewidth ~0.68 nm. **c** The lasing wavelength under various input pumping powers. The error bars in **b**, **c** express standard errors deduced by fitting. **d** Logarithmic *L*–*L* plot of the PC laser. Dots show the experimental data and the solid lines are theoretically calculated results for various values of *β* by using rate equation analyses. **e** Normalised PL spectra from representative PC lasers above lasing threshold.

The measured spectra under various pumping powers of a single mode PC laser with *a* = 310 nm and *r*/*a* *=* 0.27 are shown in Fig. 3a, in which the lasing peak locates in the ground state. The normalised frequency of measured fundamental mode lasing peak (~0.237) is slightly different from the calculated value (0.236), mainly caused by the fabrication fluctuations. The collected intensity (*L–L*) and the linewidth of the lasing peak at ∼1306 nm under various pumping powers are shown in Fig. 3b, which exhibits the evidence of the lasing with a clear kink of *L–L* curve and the spectral linewidth narrowing effect. The lasing threshold is estimated to be around 0.6 µW from the *L–L* curve. The inset in Fig. 3b shows the Lorentzian curve fitting of measured data just below the threshold, which indicates a linewidth ~0.68 nm and a cavity *Q*-factor (*Q* = *λ*/Δ*λ*) around 2177. Figure 3c displays a red-shift of the measured lasing peak with increasing incident pumping powers mainly induced by thermal effects. A red-shift with *dλ/dP*_pump_ ~ 25 nm/mW is obtained using a linear fit. The *L–L* curve in Fig. 3b shows a soft turn-on of the laser operation, which is typical behaviour for a laser with high spontaneous emission coupling efficiency *β*^48^. In order to evaluate the *β* of the fabricated PC nanocavity laser, the experimental *L–L* plot is compared with theoretical curves calculated by using coupled rate equations. Carrier density (*N*) and photon density (*P*) in the cavity are described by the following conventional rate equation model^49^:

1 $$\frac{{dN}}{{dt}} = \eta \frac{{P_{in}}}{{\hbar \omega _pV_a}} - \frac{N}{{\tau _r}} - \frac{N}{{\tau _{nr}}} - v_gg\left( N \right)P$$

2 $$\frac{{dP}}{{dt}} = \Gamma v_gg\left( N \right)P + \Gamma \beta \frac{N}{{\tau _r}} - \frac{P}{{\tau _P}}$$

where *η* is the absorption ratio of the pump laser in the active region, *ω*_*p*_ is the frequency of the pump laser, *V*_*a*_ is the active volume, and the *τ*_*r*_ (*τ*_*nr*_) is the radiative (non-radiative) recombination lifetime. The non-radiative lifetime *τ*_*nr*_ is too long to significantly affect the fitting results compared with *τ*_*r*_. We also expect that the non-radiative surface recombination occurring at the etched surface is significantly lower than in quantum well lasers, as a result of spatially confinement of InAs quantum dot exciton^50^. The *v*_*g*_ is the group velocity. The *Γ* is the confinement factor, of which a value of 0.16 is estimated from the intersection of the mode volume with the four-stacked InAs/GaAs QDs. A logarithmic gain function *g(N)* *=* *g*_*0*_*log(N*/*N*_*tr*_*)* is assumed, where *N*_*tr*_ is the transparency carrier density. The photon lifetime *τ*_*p*_ is represented as follows: *τ*_*p*_ = *λQ*/2*πc*, where *λ* is the lasing wavelength and *Q* is the quality factor of the lasing mode. As shown in the Fig. 3d, the best fit to the measured data is obtained with *β* = 0.18, and *N*_*tr*_ = 9.4 × 10^15^ cm^−3^ for the demonstrated PC laser. In addition, the normalised spectra of various PC lasers above threshold with slightly different radius of air-holes and lattice constant are shown in Fig. 3e, of which a wide tunable range near 70 nm is achieved by changing the structural parameters.

**Fig. 4:**
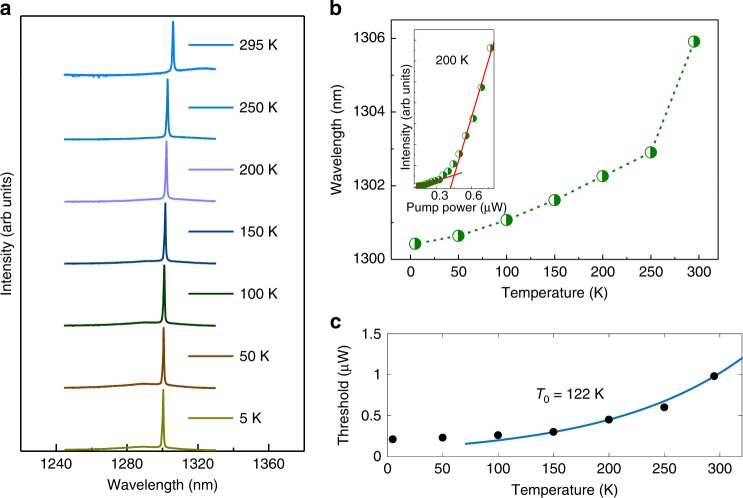
Temperature dependent laser performance. **a** Normalised lasing spectra at the pumping power 1.6 μW (above threshold) from 5 to 295 K. **b** Temperature dependence of lasing wavelength at the pumping power 1.6 μW, the inset shows a measured *L*–*L* curve at 200 K. **c** Temperature dependence of the lasing threshold, and the blue line represents the exponential fit to the experimental data (black dot).

Temperature dependent PL spectra of the optically pumped PC lasers are measured to characterise the thermal stability of InAs QD PC lasers, as shown in Fig. 4. Figure 4a depicts the normalised single-mode lasing spectra at the pumping power 1.6 μW (above threshold) from 5 to 295 K, with structural parameters *a* = 320 nm and *r*/*a* = 0.3. The lasing wavelength presents a red-shift with increased temperature as shown in Fig. 4b, resulting from the InAs bandgap shrinkage and temperature-induced cavity effective index change^51^. The inset in Fig. 4b presents the measured *L–L* curve of the PC laser at 200 K, indicating a threshold around 0.45 μW. The lasing thresholds as a function of temperature in Fig. 4c indicates that the thresholds are increased by a factor of ∼5 as the temperature increases from 5 to 295 K, which can be attributed to the enhanced nonradiative recombination and the restrained carrier confinement in the QD active region. The measured thresholds from 100 to 295 K can be fitted with an exponential function using *P*_*th*_ ∝ *exp*(*T*/*T*_0_)^52^, and the characteristic temperature *T*_*0*_ is extracted to be around 122 K.

**Fig. 5:**
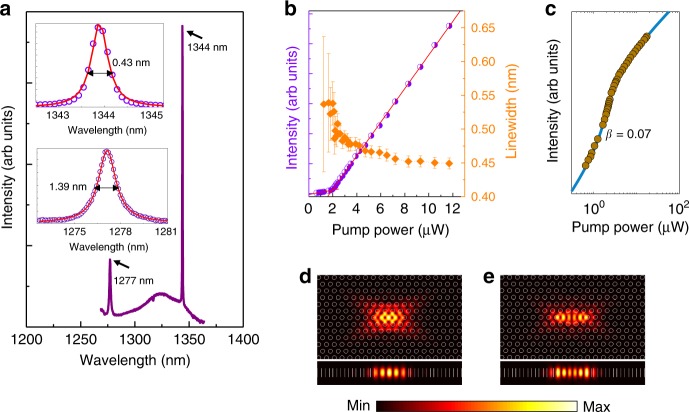
Lasing performance with different structural parameters. **a** Collected lasing spectrum above threshold of a fabricated PC laser with lattice constant *a* *=* 315 nm and radius *r*/*a* = 0.27. The inset shows the magnified PL spectra of the lasing peak at 1344 nm and the resonant peak at 1277 nm with the pumping power 20 μW. **b** Corresponding collected intensity and linewidth as a function of input pumping powers for the emission peak at 1344 nm with lasing threshold at 1.9 μW. The error bars express standard errors deduced by fitting. **c** Corresponding logarithmic *L*–*L* plot of the PC laser, and the blue line was theoretically obtained results by using rate equation analyses. **d**, **e** present the top-view and cross-section view of the calculated E-field profiles for the fundamental mode (1344 nm) and the first higher order mode (1277 nm), respectively.

In addition to the single mode lasing at the ground state, a higher order resonant mode in the first excited state is also observed for some PC lasers, as shown in Fig. 5. Figure 5a depicts the measured lasing spectrum above threshold of a fabricated PC laser with lattice constant *a* = 315 nm and radius *r*/*a* = 0.27. The upper inset in Fig. 5a presents a magnified PL spectrum of a lasing peak at 1344 nm (fundamental mode) under a pumping power of 20 μW, showing a linewidth around 0.43 nm above the threshold. The bottom inset depicts the resonant peak at 1277 nm with a wider linewidth around 1.39 nm under the same pumping power. The corresponding collected intensity and linewidth as a function of input pumping powers for the emission peak at 1344 nm are shown in Fig. 5b, which indicates a lasing threshold at 1.9 μW with a fitted *β* = 0.07 shown in Fig. 5c. Figure 5d, e present the top-view and cross-section view of the calculated electric field (E-field) profiles for the fundamental mode (1344 nm) and the first higher order mode (1277 nm), respectively. While increasing the pumping power, the resonant peak at 1277 nm fails for lasing with intensity much weaker than the fundamental mode, even by increasing the pumping power to a high level. However, for PC cavity with higher order mode locates near the central wavelength of the excited state, lasing emission is observed by increasing the pumping power due to the gain switching (Supplementary Fig. 3). And a single mode lasing in the excited state can be achieved by modifying the lattice constant or radius of etched air-holes (Supplementary Fig. 4).

## Discussion

In conclusion, we report the first demonstration of ultrasmall InAs/GaAs QD PC membrane lasers monolithically grown on CMOS-compatible Si substrates. The PC laser with a small mode volume of 0.88 (*λ*/*n)*^3^ was CW optically pumped at room temperature and exhibits an ultra-low lasing threshold of ∼0.6 µW, as well as a large spontaneous emission coupling efficiency up to 18% evidenced by rate equation analysis. In addition, a high characteristic temperature of *T*_*0*_ ∼ 122 K is extracted through the exponential fit of threshold as a function of temperature from 100 to 295 K. The demonstrated Si-based PC lasers with a small footprint as well as a low power consumption are expected to play an important role in the next-generation nanoscale Si photonics.

## Method

### Growth of photonic crystal lasers on Si

The InAs/GaAs QD PC membrane lasers were grown on planar on-axis Si (001) substrates. To overcome the antiphase boundaries (APBs) problem, a two-step 400 nm of APB-free epitaxial GaAs film was first deposited on a pre-treated bi-atomic Si (001) substrate with 300 mm diameter, by using metal-organic chemical vapour deposition^53^. Then the GaAs/Si wafer was covered with a layer of photoresistor for dicing to 2-inch wafers and transfer to molecular beam epitaxial growth. The photoresistor is removed by using acetone in a ultrasonic cleaner before moving into the MBE chamber. A 200 nm GaAs buffer layer has been grown on the on-axis (001) GaAs/Si substrate to achieve a smooth surface at 590 °C, which is examined by a clear reflective high energy electron diffraction 4 × 2 pattern. Four sets of DFLs have been used to suppress the propagation of threading dislocations. Each set of DFLs includes five sets of In_0.18_Ga_0.82_As/GaAs strained-layer superlattices grown at 480 °C and a 300 nm GaAs spacing layer grown at 590 °C^54,55^. The active region with 4 layers of InAs/GaAs DWELL has been grown between the upper and lower 40 nm Al_0.4_Ga_0.6_As cladding layer grown at 600 °C, which are grown on the top of 1 μm Al_0.6_Ga_0.4_As sacrificial layer. Each layer of DWELL consists of three monolayers of InAs deposited on a 2 nm In_0.15_Ga_0.85_As quantum well and capped by a 6 nm In_0.15_Ga_0.85_As layer at 510 °C, which separated by a 50 nm GaAs spacing layer grown at 590 °C.

### Photonic crystal lasers fabrication

First, a layer of SiO_2_ with a thickness of ~120 nm was deposited on the as-grown wafer by plasma-enhanced chemical vapor deposition (PECVD) as a hard mask for PC dry etching. A 220 nm ZEP520 electron beam resist thin film was spin coated on the surface of the hard mask. Subsequently, the electron beam lithography was used to define the PC pattern in ZEP520. The PC pattern was transferred from ZEP520 into the hard mask using reactive ion etching (RIE). Afterwards, the electron beam resist was removed by using RIE with O_2_ plasma. Then chlorine-based inductively coupled plasma RIE (ICP-RIE) dry etching was performed subsequently to obtain the air-holes through the active region and the sacrificial layer. The recipe of the ICP-RIE dry etching was based on a mixture of Cl_2_ and N_2_, with flow rates at 12 and 5 sccm, respectively. Other key parameters of ICP-RIE dry etching include 75 W bias power and 320 W ICP power, an elevated substrate temperature of 40 °C, chamber pressure at 3 mTorr and etching time of 50 s. The residual SiO_2_ hard mask was removed in the diluted hydrofluoric acid. Finally, the sacrificial layer Al_0.6_GaAs with thickness of ∼1 µm was wet-etched by immersing the fabricated sample in 40 % hydrofluoric acid solution for 30 s to form an air region slab underneath the PC active slab. The air region surrounding the PC cavity can efficiently enhance the light confinement in the vertical direction.

### PL measurements

The fabricated PC lasers were CW optically pumped with a micro-photoluminescence (µ-PL) measurement system in a surface-normal pump configuration, using a CW 632.8 nm He-Ne laser as the excitation source. The focused laser spot was positioned on the centre region of the fabricated PC cavity using piezo-electric nanopositioners and its size was estimated to be ∼2.5 µm in diameter by using an ×100 objective. The emission spectra were collected from the top by using the same objective and analysed by a monochrometre with a thermoelectric-cooled InGaAs detector. Long-pass filters were used to block the excitation light from reaching the detector. For temperature dependent measurements, the sample was mounted in a helium gas flow cryostat, with device temperature controlled from 5 to 295 K.

### Reporting summary

Further information on research design is available in the Nature Research Reporting Summary linked to this article.

## Supplementary information

Supplementary Information

Reporting Summary

## Data Availability

The data that support the findings of this study are available from the corresponding author upon reasonable request.
