# Supplementary material for: Continuous-wave quantum dot photonic crystal lasers grown on on-axis Si (001)
Source: Nat Commun. 2020 Feb 20;11:977. doi: 10.1038/s41467-020-14736-9 (PMC7033092; doi:10.1038/s41467-020-14736-9)
Supplement: Supplementary file 1 — Supplementary Information [file 41467_2020_14736_MOESM1_ESM.pdf]

**Supplementary information for**

# Continuous-Wave Quantum Dot Photonic Crystal Lasers Grown on On-axis Si (001)

**T. Zhou et al**

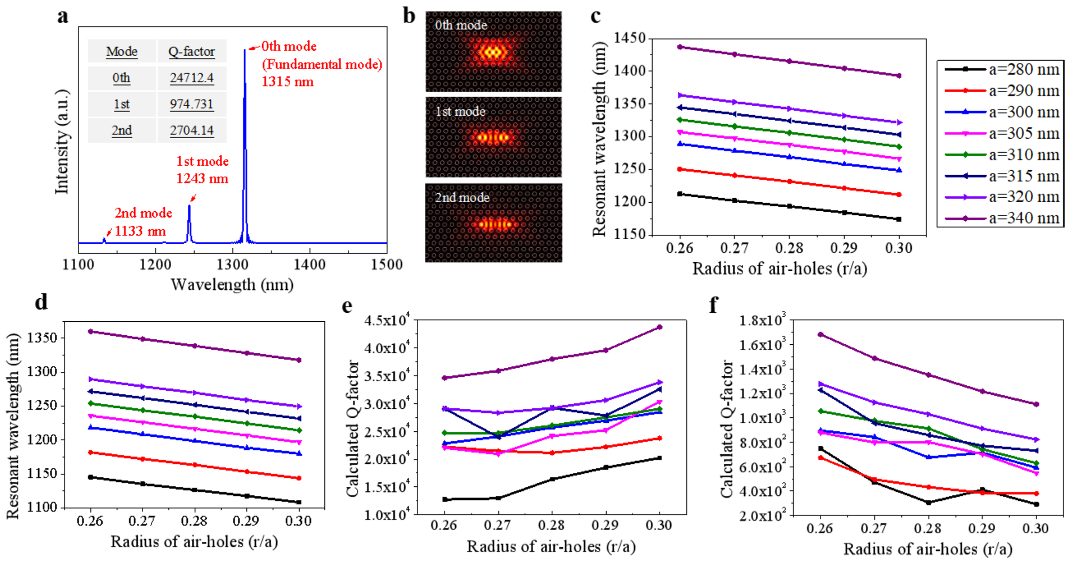


**Supplementary Figure 1 | Device simulation. a** Calculated spectra of L3 photonic crystal cavity by using 3D-FDTD method, with lattice constant *a* = 310 nm, *r/a* = 0.27. Three main resonant modes are labeled in the spectra with inset showing the calculated *Q*-factors. The fundamental mode (0th mode) has highest *Q*-factor. **b** Corresponding electric-field profiles of three main modes within photonic crystal cavity. **c** and **d** show the calculated resonant wavelength of the fundamental mode and the first higher order mode of the L3 defect photonic crystal cavity under various lattice constant and radius of etched air-holes, respectively. Corresponding calculated *Q*-factor of the fundamental mode **e** and the first higher order mode **f**.

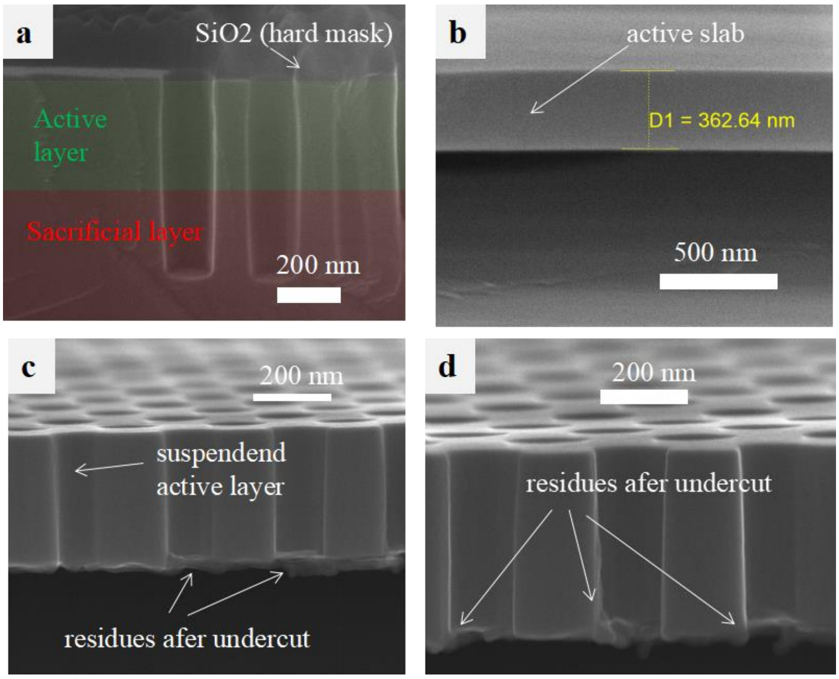


**Supplementary Figure 2 | Device fabrication. a** Etching profile of air-holes before wet-etching of the sacrificial layer, showing smooth sidewall of air-holes. **b** Undercut profile of a suspended active layer. **c** and **d** present a suspended photonic crystal slab, which indicates some residues remain on the undercut surface.

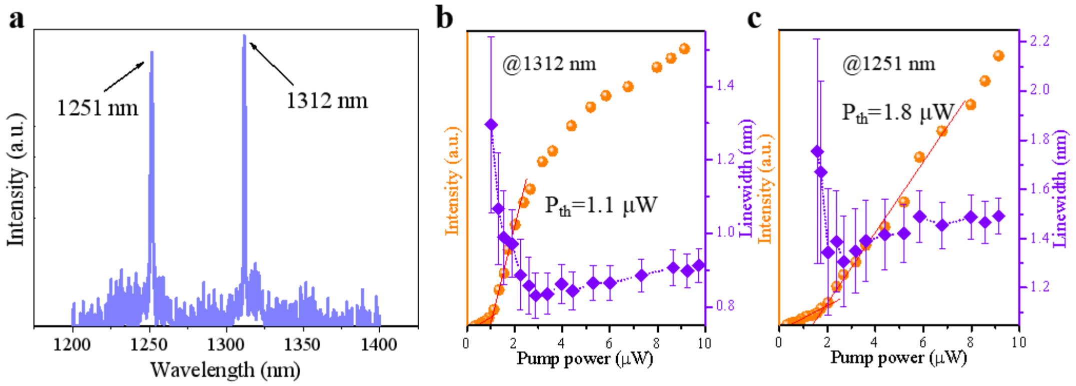


**Supplementary Figure 3 | Multi-mode lasing of a PC laser. a** Measured multi-mode lasing spectra showing lasing peaks locate at the ground state and the first excited state, the lattice constant is $a$ = 315 nm, $r/a$ = 0.29. **b** and **c** showing the collected L-L curve and linewidth under various pump powers of lasing peak at 1312nm and 1251 nm, respectively.

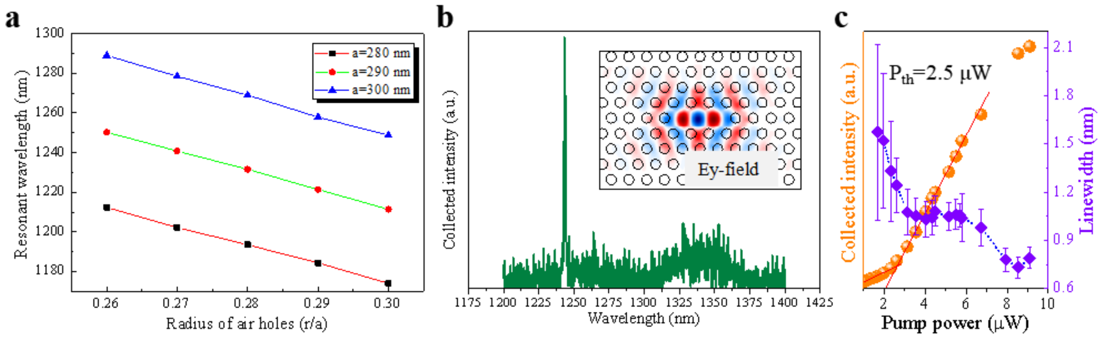


**Supplementary Figure 4 | Single mode lasing in the excited state.** **a** Calculated resonant wavelength of fundamental mode for L3 photonic crystal cavity by using 3D-FDTD methods, the lattice constant *a* is ranging from 280 nm to 300 nm, and radius *r/a* = 0.26 to 0.3. **b** presents the lasing spectra above the threshold with structural parameter *a* =290 nm, *r/a* = 0.27, and the inset shows Ey profile of the fundamental mode, and **c** illustrates the corresponding L-L curve and linewidth under various pump powers.

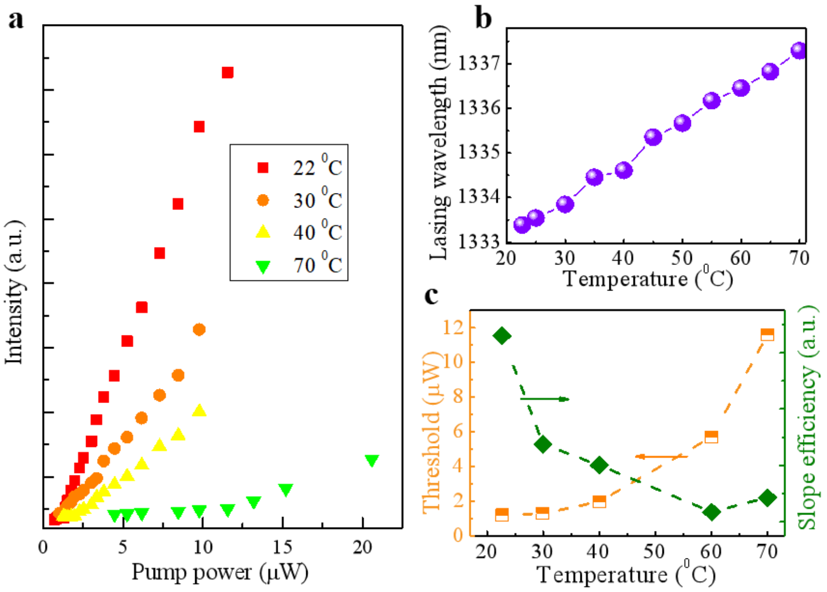


**Supplementary Figure 5 | Laser performance above room-temperature. a** Collected temperature dependent *L*-*L* curves from 22 °C to 70 °C. **b** The lasing wavelength at fourth the threshold under various temperature. **c** Temperature dependence of lasing thresholds and the relative slope efficiency of the lasing peak.
