# Supplementary material for: Continuous-wave quantum dot photonic crystal lasers grown on on-axis Si (001)
Source: Nat Commun. 2020 Feb 20;11:977. doi: 10.1038/s41467-020-14736-9 (PMC7033092; doi:10.1038/s41467-020-14736-9)
Supplement: Supplementary file 2 — Reporting Summary [file 41467_2020_14736_MOESM2_ESM.pdf]

natureresearch

Corresponding author(s): Huiyun Liu

Lasing Reporting Summary

Nature Research wishes to improve the reproducibility of the work that we publish. This form is intended for publication with all accepted papers reporting claims of lasing and provides structure for consistency and transparency in reporting. Some list items might not apply to an individual manuscript, but all fields must be completed for clarity.

For further information on Nature Research policies, including our data availability policy, see Authors & Referees.

ü Experimental design

Please check: are the following details reported in the manuscript?

1. Threshold

Plots of device output power versus pump power over a wide range of values indicating a clear threshold

[x] Yes [ ] No

This information can be found in Fig. 3(b), Fig. 4(b) and Fig. 5(b).

2. Linewidth narrowing

Plots of spectral power density for the emission at pump powers below, around, and above the lasing threshold, indicating a clear linewidth narrowing at threshold

[x] Yes [ ] No

This information can be found in Fig. 3(b) and Fig. 5(b).

Resolution of the spectrometer used to make spectral measurements

[x] Yes [ ] No

The resolution of the spectrometer is around 0.1nm, which is lower than the laser linewidth.

3. Coherent emission

Measurements of the coherence and/or polarization of the emission

[ ] Yes [x] No

Our experimental setup does not allow for measurements of the coherence and polarization information.

4. Beam spatial profile

Image and/or measurement of the spatial shape and profile of the emission, showing a well-defined beam above threshold

[ ] Yes [x] No

Beam spatial profile was not collected in this stage, limited by the measurement set-up.

5. Operating conditions

Description of the laser and pumping conditions
Continuous-wave, pulsed, temperature of operation

[x] Yes [ ] No

Continuous-wave pumping, room-temperature operation. And the pumping conditions are also described in the Methods section.

Threshold values provided as density values (e.g. W cm^{-2} or J cm^{-2}) taking into account the area of the device

[x] Yes [ ] No

Considering the approximated area of L3 photonic crystal cavity to be 4a*sqrt(3)a (a is the lattice constant), the threshold is 90.1 W/cm-2 for the demonstrated laser in Fig. 3, 133.9 W/cm-2 for the demonstrated laser operating at 295K in Fig. 4, and is 276.4 W/cm-2 for the demonstrated laser in Fig. 5.

6. Alternative explanations

Reasoning as to why alternative explanations have been ruled out as responsible for the emission characteristics
e.g. amplified spontaneous, directional scattering; modification of fluorescence spectrum by the cavity

[x] Yes [ ] No

We achieve lasing spectra with narrow linewidth ~0.7nm above threshold (as shown in Fig. 3 and Fig. 4), and a lasing peak with linewidth ~0.43 nm (see Fig. 5(a)) was achieved. In addition, L-L curve and linewidth narrowing (see Fig. 3(b), Fig. 4(b) and Fig. 5(b)) provide the evidence for lasing emission. Therefore, there are not other explanations (such as amplified spontaneous) as responsible for the emission characteristics.

7. Theoretical analysis

Theoretical analysis that ensures that the experimental values measured are realistic and reasonable
e.g. laser threshold, linewidth, cavity gain-loss, efficiency

[x] Yes [ ] No

The thresholds are determined by the L-L curve, and linewidth is obtained by using Lorentzian curve fitting of the lasing peak.

8. Statistics

Number of devices fabricated and tested	☒ Yes<br>☐ No	We fabricated arrays of photonic crystal lasers with slightly different lattice constant and radius of air-holes, and test the fabricated devices. In the revised version, we demonstrated typical lasing spectra of 11 devices (see Fig. 3(a), Fig. 3(e), Fig. 4(a) and Fig. 5(a)), and 2 devices in the Supporting Information (see Fig. S3 and Fig. S4).
Statistical analysis of the device performance and lifetime (time to failure)	☐ Yes<br>☒ No	The emission properties are stable and lifetime to failure is not measured limited by the measurement setup.
